# Comparison of clinical outcomes and prognosis between surgery and endoscopic submucosal dissection in patients with synchronous multifocal early gastric cancer

**DOI:** 10.1186/s12893-023-02194-1

**Published:** 2023-09-26

**Authors:** Linsen Bao, Hengfei Gao, Lingxiao Pu, Chao Sui, Kangkang Ji, Feng Wang, Liang Tao, Min Feng, Meng Wang

**Affiliations:** 1 Department of General Surgery, Nanjing Drum Tower Hospital, Affiliated Hospital of Medical School, Nanjing University, Nanjing, China; 2 Medical School of Nanjing University, Nanjing, China

**Keywords:** Synchronous multiple neoplasms, Endoscopic submucosal dissection, Surgical treatment, Prognosis

## Abstract

**Background:**

Synchronous multiple early gastric cancer (SMEGC) refers to the simultaneous occurrence of two or more malignant cancer lesions in the stomach. For patients with multiple early gastric carcinomas, the choice of appropriate treatment remains controversial. This study is dedicated to comparing the clinical outcomes and prognosis of patients with SMEGC who underwent endoscopic submucosal dissection (ESD) or gastrectomy.

**Methods:**

A total of 180 patients with more than one malignant cancer lesion in the stomach who had received gastrectomy or ESD between 2012 and 2021 were retrospectively evaluated to determine their clinical outcomes and prognosis. Univariate and multivariate logistic regression were utilized to identify risk factors for tumor recurrence.

**Results:**

Over the 57.5 months median follow-up period for the 140 enrolled cases, tumor recurrence occurred in 8 (12%) in the ESD group but only 1 (1%) in the surgery group. Relapse-free survival (RFS) was higher in the surgery group (p = 0.023) in all cases; however, there was no significant difference in Overall survival (OS, p = 0.772). Complications were significantly higher in the surgery group than in the ESD group, but fewer in the radical distal gastrectomy group. Multivariate regression analysis revealed that ESD(p = 0.034), the main lesion size > 2 cm(p = 0.019), and undifferentiated tumor(p = 0.022) were independent risk factors for tumor recurrence.

**Conclusions:**

For the treatment of simultaneous multifocal early gastric cancer, ESD has a good short-term effect and higher quality of life. However, ESD has a higher risk of recurrence than surgery. And we found that the partial gastrectomy appears to be considered as adequate treatment for some SMEGC patients.

## Introduction

Gastric cancer remains a leading malignant tumor in China. Its incidence rate ranks third among all tumors, and the mortality rate ranks third [1]. Currently, with advances in endoscopic techniques and the popularity of gastric cancer screening, the detection rates of early gastric cancer (the depth of tumor invasion was limited in mucosa and submucosal regardless of lymph node status [2]) and gastric dysplasia has increased in China. When more than one malignant lesion, including high grade dysplasia is found during endoscopy, the patient is diagnosed as having synchronous multiple early gastric cancer (SMEGC) [3]. According to previous literature reports, the incidence rate of multiple gastric early cancer ranges from 6 to 14% in gastric cancer [3–6]. Previous studies have shown that SMEGC has several characteristics, including occurring in older men, well-differentiated tumors, early-stage tumors, and does not increase the risk of lymph node metastasis [7]. The prognosis of SMEGC is similar to that of early gastric cancer of a single lesion.

Endoscopic submucosal dissection (ESD) as a minimally invasive method with less trauma, fewer complications, and similar therapeutic efficacy, has been widely accepted. According to the Guidelines for endoscopic diagnosis of early gastric cancer, the absolute indications for ESD are greater in number than before [8], and ESD become a major treatment for early gastric cancer without lymph node metastasis. But it is not clear whether multifocal early gastric cancer applies to this standard. Most current studies focus on the comparison of prognosis between multifocal gastric cancer and unifocal gastric cancer under ESD treatment [5, 9].In this research, we aimed to compare the clinical outcomes and prognosis of ESD and gastrectomy in patients with SMEGC in patients, and compare the advantages and disadvantages of the formal anatomic resection versus ESD.

## Methods and materials

### Patients

Between 2011 and 2021, all patients diagnosed with SMEGC in our hospital were collected. The inclusion criteria included:1) postoperative pathology confirmed 2 or more tumor or dysplasia lesions;2) each lesion must have distinct demarcation from adjacent areas of the microscopically normal gastric wall or transformation zone;3) each is not caused by local extension or metastasis of another lesion [10]; 4) postoperative pathology confirmed that the depth of tumor invasion was limited in mucosa and submucosal regardless of lymph node status; the exclusion criteria included:1) patients with other malignant tumor history; 2) with preoperative chemotherapy;3) pathological or clinical data was not complete.

### Definitions

Curative resection was defined as complete resection with negative horizontal and vertical margins, with no nerve or lympho-vascular invasion [11].

In the statistical sequence of lesions in patients was also defined, the lesion with the largest depth of tumor invasion was identified as the main lesion; and if two or more tumor lesions have the same depth of invasion, the largest lesion is considered as the main lesion, the remaining tumor lesions regarded as the minor lesions [12]. The maximum diameter of tumor was measured by the postoperative specimen.

Synchronous recurrence was defined as a newly developed adenocarcinoma or high-grade dysplasia within 12 months after curative resection, while metachronous recurrence was defined as a newly developed adenocarcinoma or high-grade dysplasia which had developed more than 12 months after curative resection. Our hospital provides eradication therapy for all patients diagnosed with H. pylori infection upon discharge.

### Data collection

All clinical data, including demographics, pathological results, gastrectomy types and postoperative complications, were collected from the clinical database.

### Statistical analysis

SPSS version 26.0 statistical software was used for statistical analysis. All continuous variables were described as mean ± standard deviation (SD) or median (interquartile range, IQR), and compared by student’s test or Mann-Whitney U test. All categorical variables were described as frequency (percentage), and the chi-square test or Fisher’s exact test was used to analyze categorical variables when applicable. The prognostic overall survival (OS) and relapse-free survival (RFS) were conducted by the Kaplan-Meier method. P-value less than 0.05 was considered statistically significant.

## Results

### Clinical baseline characteristics

**Fig. 1:**
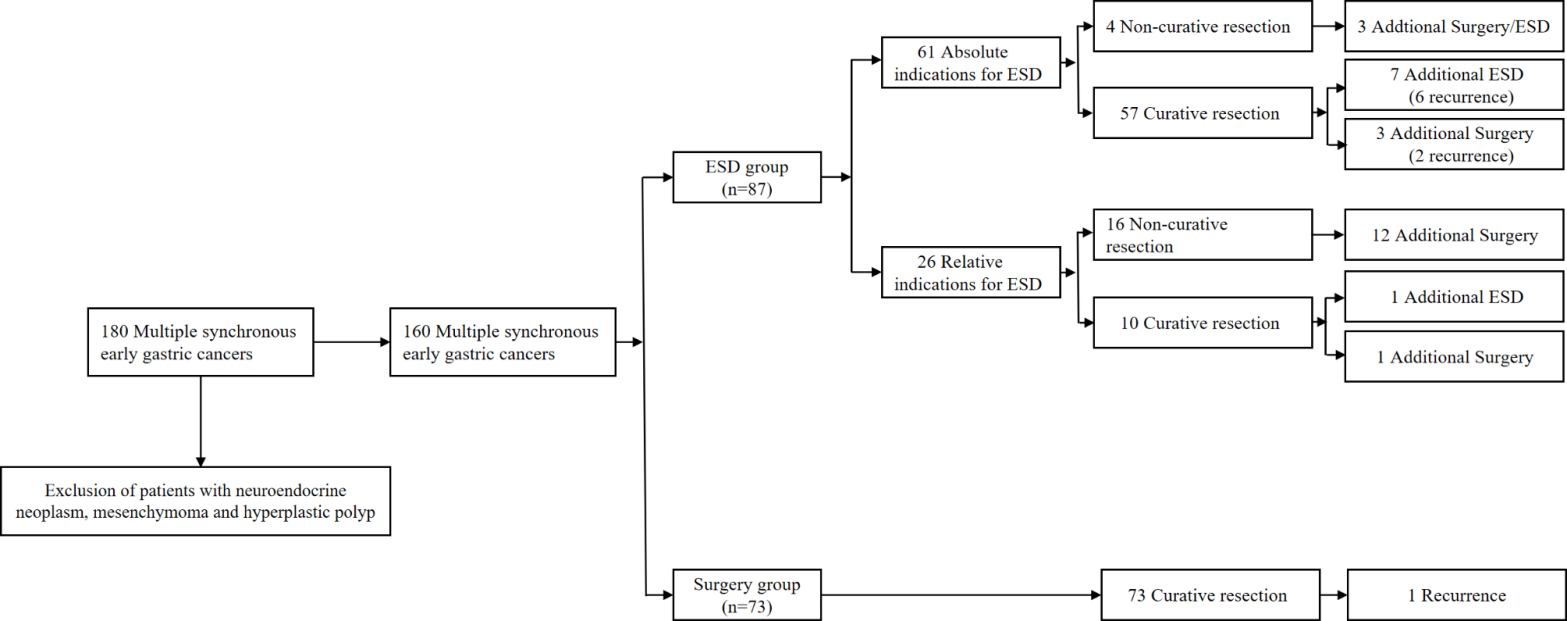
Patient selection of multiple synchronous early gastric cancers treated by ESD and surgery

**Fig. 2:**
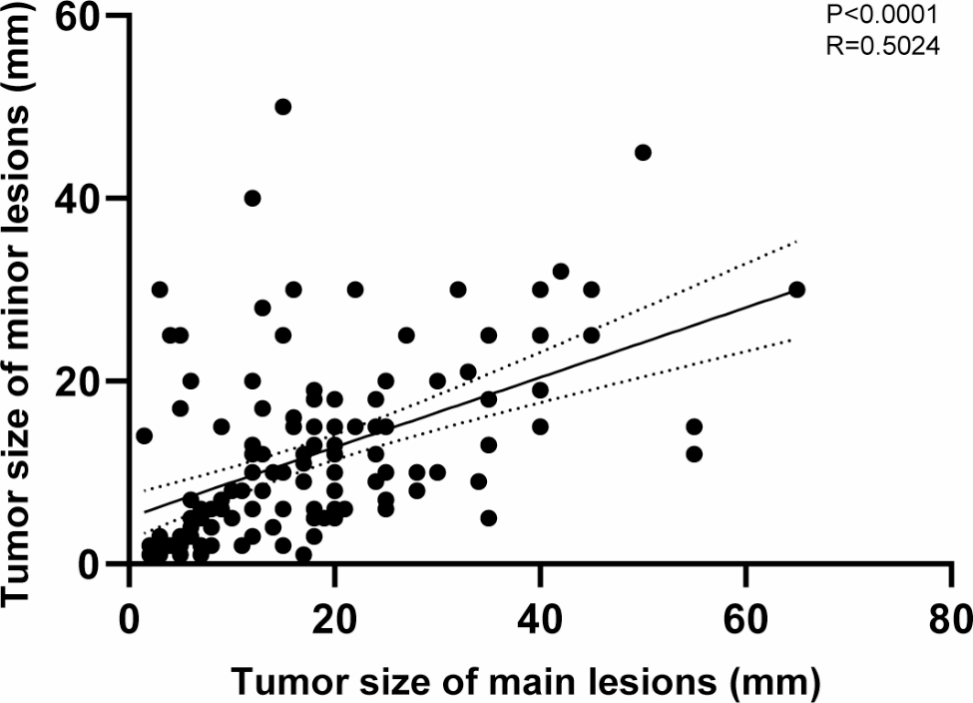
Correlation of tumor size in main and minor lesions of multiple synchronous early gastric cancers

A total of 180 patients who underwent gastrectomy or ESD therapy were included in our research,101 patients were in the ESD group and 79 patients were in the surgery group. Among them, due to the exclusion of patients with neuroendocrine neoplasm, gastrointestinal stromal tumors, and hyperplastic polyp, we were left with 87 patients in ESD group and 73 patients in surgery group, as shown in Fig. 1. After excluding patients who underwent non-curative resection, we were left with 140 patients, including the gastrectomy groups (n = 73) and the ESD group (n = 67). There were 309 independent lesions, 17 patients had 3 independent lesions, 6 patients had 4 independent lesions in the stomach, and the remaining patients had 2 independent lesions. The size of the main and minor lesions had an important positive correlation (r = 0.5024, P < 0.0001), as the size of the minor lesion increased with the increase of the main lesion (Fig. 2).

The baseline characteristics of the 140 patients are listed in Table 1. The size of the minor lesion showed that lesion larger than 2 cm was more frequent in the surgery group than in the ESD (26%vs12% P = 0.035) group. There was no significant difference in the mean age and underlying diseases. In the pathologic findings of main and minor lesions, poorly differentiated adenocarcinoma was more common in the surgery group (25%vs6%, P < 0.001;21%vs6%, P < 0.001 respectfully). Ulcerated lesions were more frequent in the surgery group than in the ESD group (38%vs8%, P < 0.001) as was the invasion of the submucosa (13%vs33%, P = 0.007).

**Table 1:** Baseline characteristics of the patients who underwent curative ESD or Surgery

Variables	Total(n = 140)	ESD(n = 67)	Surgery(n = 73)	P value
Age, years				
Mean ± SD		68.4 ± 8.3	67.2 ± 10.2	0.467
Sex, n%				0.433
Male	111	55 (82)	56 (77)	
Female	29	12 (18)	17 (24)	
Underlying disease, n (%)				
Hypertension	51	28 (42)	23 (32)	0.207
Diabetes mellitus	11	8 (12)	3 (4)	0.085
Cardiac diseases	12	5 (8)	7 (10)	0.653
Cerebral diseases	4	2 (3)	2 (3)	1.000
Liver cirrhosis	5	3 (5)	2 (3)	0.580
Chronic kidney disease	0	0 (0)	0 (0)	0
Location (a) n (%)				0.648
Upper	45	21 (31)	24 (33)	
Middle	29	12 (18)	17 (23)	
Lower	66	34 (51)	32 (44)	
Location (b) n (%)				0.831
Upper	28	12 (18)	16 (22)	
Middle	40	20 (30)	20 (27)	
Lower	72	35 (52)	37 (51)	
Lesion a size, mm				
Median (IQR)		1.7 (1.2–2.4)	1.5 (0.6–2.5)	0.466
Lesion b size, mm		1.0 (0.6–1.5)	1.0 (0.3-2.0)	0.482
Lesion a size, mm				0.397
<2 cm	89	45 (67)	44 (60)	
>=2 cm	51	22 (33)	29 (40)	
Lesion b size, mm				0.035
<2 cm	113	59 (88)	54 (74)	
>=2 cm	27	8 (12)	19 (26)	
Histology a, n (%)				< 0.001
Differentiated type	81	43 (64)	38 (52)	
Undifferentiated type	22	4 (6)	18 (25)	
Mixed type	9	9 (13)	17 (23)	
Dysplasia^1^	28	11 (17)	0 (0)	
Histology b, n (%)				< 0.001
Differentiated type	65	33 (49)	32 (44)	
Undifferentiated type	19	4 (6)	15 (20)	
Mixed type	10	0 (0)	10 (14)	
Dysplasia^1^	46	30 (45)	16 (22)	
Ulcer	33	5 (7)	28 (38)	< 0.001
Depth of invasion a, n (%)				0.007
Mucosa	107	58 (87)	49 (67)	
Submucosa	33	9 (13)	24 (33)	
Recurrence n (%)				
Any recurrence	9	8 (12)	1 (1)	0.014
Synchronous n (%)	4	4 (6)	0 (0)	0.018
Metachronous n (%)	5	4 (6)	1 (1)	
Family history of gastric cancer, n (%)	34	15 (22)	19 (26)	0.616
Adverse event	12	1 (2)	11(15)	0.004
Death, n (%)	3	1 (2)	2 (3)	1.000

*SD* standard deviation; *IQR* interquartile range; *ESD* endoscopic submucosal dissection;

*a* refers to main lesion; *b* refers to minor lesion;

Dysplasia^1^ includes high-grade intraepithelial neoplasia and low-grade intraepithelial neoplasia

**Table 2:** Univariate analysis and multivariate analysis of risk factors for tumor recurrence in surgery and ESD for curative resection groups

Univariate Analyze									Multivariate Analyze		
				OR	95%CI	P Value			OR	95%CI	P Value
Group (ESD)				9.763	1.187–80.296	0.034			14.753	1.619-134.461	0.017
Age (≥ 65)				0.683	0.184–2.538	0.449					
Sex(female)				0		0.998					
Lesion a(≥ 2 cm)				6.920	1.380-34.717	0.019			9.347	1.665–52.485	0.011
Lesion b(≥ 2 cm)				1.212	0.237–6.188	0.818					
Ulcer (+)				0.387	0.047–3.211	0.379					
Location (a, Middle)				0.232	0.026–2.037	0.118			0.214	0.021–2.213	0.196
Location (a, Lower)				0.206	0.040–1.074	0.061			0.167	0.028–1.005	0.051
Location (b, Middle)				1.054	0.164–6.759	0.956					
Location (b, Lower)				0.799	0.134–4.497	0.777					
Histology (a, undifferentiated carcinoma)				1.057	0.204–5.490	0.947					
Histology (a, mixed-type carcinoma)				0	0	0.999					
Histology (a, dysplasia^1^)				0	0	0.998					
Histology (b, undifferentiated carcinoma)				3.706	0.486–28.269	0.206					
Histology (b, mixed-type carcinoma)				0	0	0.999					
Histology (b, dysplasia^1^)				3.841	0.711–20.742	0.118					
Depth of invasion(T1b)				0	0	0.998					

*ESD* endoscopic submucosal dissection; *a* refers to main lesion; *b* refers to minor lesion;

Dysplasia^1^ includes high-grade intraepithelial neoplasia and low-grade intraepithelial neoplasia

**Table 3:** Univariate analysis and multivariate analysis of risk factors for tumor recurrence in ESD for curative resection group

Univariate Analyze									Multivariate Analyze		
				OR	95%CI	P Value			OR	95%CI	P Value
Age (≥ 65)				0.405	0.019–1.807	0.236					
Sex(female)				0		0.999					
Lesion a(≥ 2 cm)				8.062	1.473–44.139	0.016			6.526	0.876–48.623	0.067
Lesion b(≥ 2 cm)				1.061	0.133–9.958	0.959					
Ulcer (+)				0		0.999					
Location (a, Middle)				0.291	0.030–2.845	0.289			0.154	0.009–2.565	0.192
Location (a, Lower)				0.200	0.035–1.147	0.071			0.058	0.003–1.187	0.065
Location (b, Middle)				0.556	0.068–4.568	0.585					
Location (b, Lower)				0.645	0.102–4.066	0.641					
Histology (a, undifferentiated carcinoma)				6.167	0.725–52.468	0.096			63.492	1.821-2213.52	0.022
Histology (a, mixed-type carcinoma)				0	0	0.999					
Histology (a, dysplasia^1^)				0	0	0.999					
Histology (b, undifferentiated carcinoma)				15.50	1.37-175.383	0.027			2.965	0.359–24.467	0.313
Histology (b, mixed-type carcinoma)				2.385	0.404–14.078	0.337					
Histology (b, dysplasia^1^)				0	0	0.999					
Depth of invasion(T1b)											

*ESD* endoscopic submucosal dissection; *a* refers to main lesion; *b* refers to minor lesion;

Dysplasia^1^ includes high grade intraepithelial neoplasia and low-grade intraepithelial neoplasia

**Table 4:** Adverse events of ESD and different surgical methods in cohort

Method	ESD	total gastrectomy	partial gastrectomy
Adverse event	1	10	1
Normal	66	33	29

### Clinical prognostic outcomes

According to the Guidelines for endoscopic diagnosis of early gastric cancer [8], patients undergoing endoscopic resection are divided into two groups: absolute indications and relative indications. There were 61 patients in the absolute indications group, 4 of whom had a non-curative resection; of these 4 patients, 1 patient received additional radical gastrectomy immediately after ESD, 1 patient was found to have differentiated adenocarcinoma 15 months after ESD and received surgical treatment, 1 patient received additional ESD during postoperative review, and no tumor recurrence occurred in the last patient during long-term endoscopic follow-up. In relative indications group, without a curative resection, 12 of whom underwent a later gastrectomy while the other 4 patients were managed by long-term endoscopic follow-up without tumor recurrence.

**Fig. 3:**
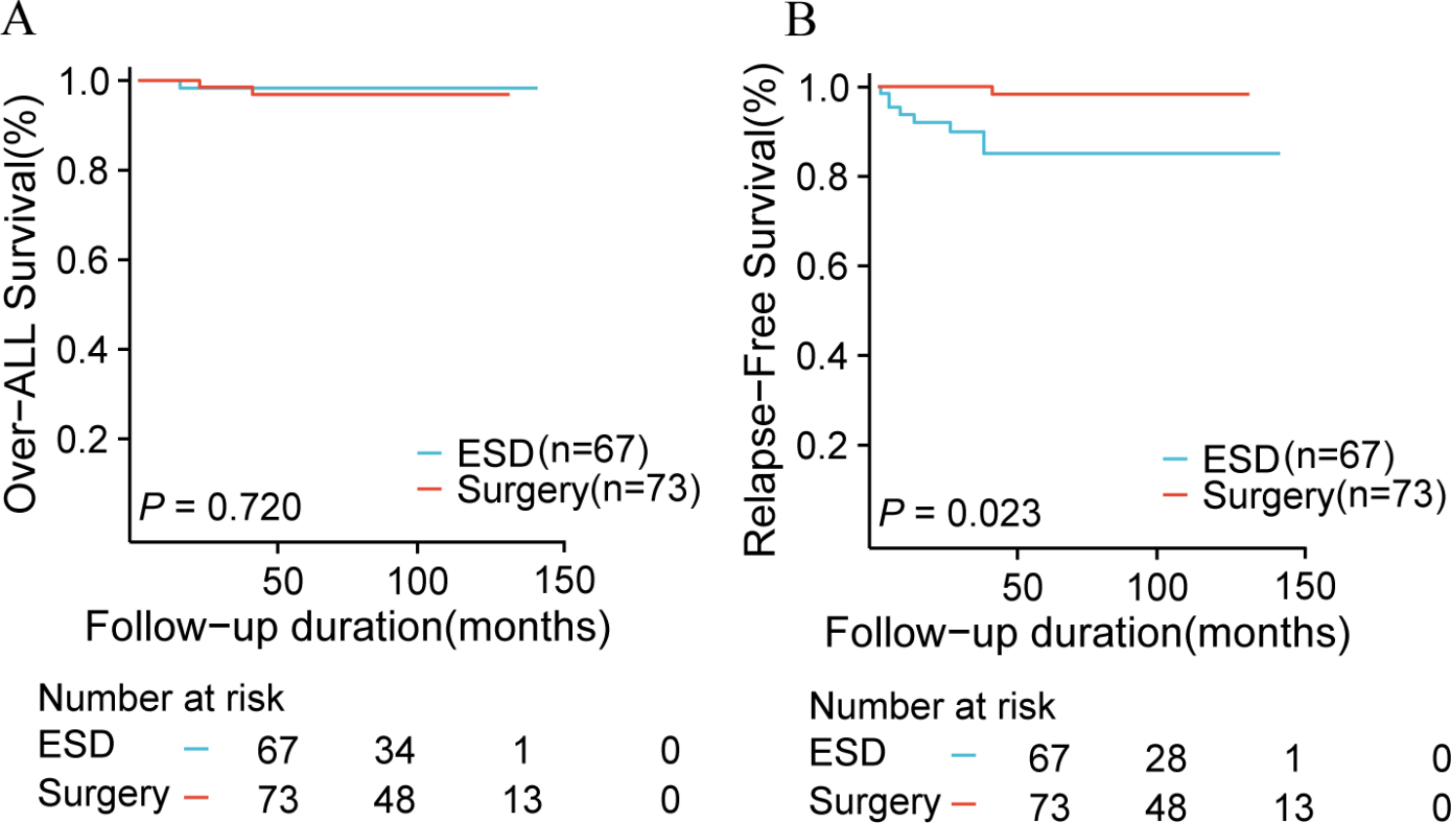
Kaplan–Meier curve comparisons of overall survival (OS) and relapse free survival (RFS) in the ESD group(n = 67) and surgery group(n = 73), **(A)** OS; **(B)** RFS, *ESD* endoscopic submucosal dissection

**Fig. 4:**
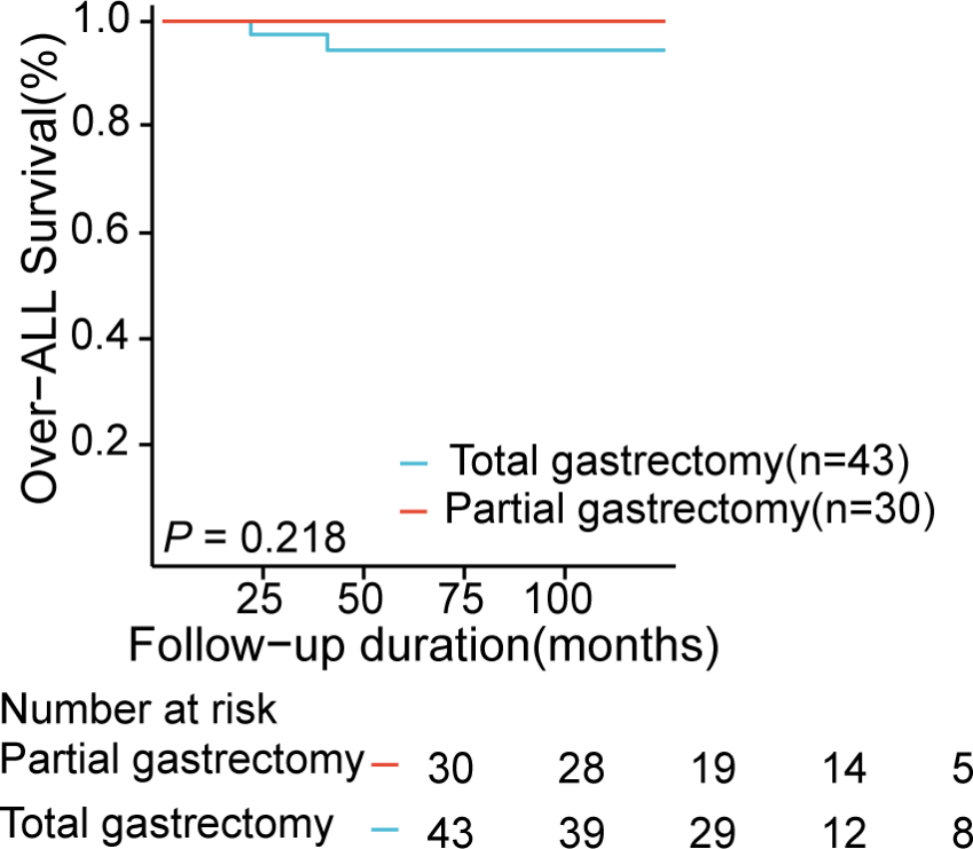
Kaplan–Meier curve comparisons of overall survival (OS) in the partial gastrectomy group (n = 30) and the total gastrectomy group (n = 43)

All the patients in the surgery group underwent curative resections, and only 1 case had tumor recurrence after surgery and died. But there were 11 cases of complications, including 2 bowel obstructions,1 anastomotic leak, and 8 developed malnutrition after gastrectomy. All 8 malnourished patients had undergone total gastrectomy, including 1 death. In the group of 67 cases undergoing an ESD for curative resection, additional radical gastrectomy and ESD were performed in 4 and 8 of these cases, respectively. Among them, tumor recurrence was confirmed in 8 cases by postoperative pathology. According to the time of recurrence, synchronous recurrences arose in 4 patients, and metachronous recurrences were detected in 4 other patients. Only 1 patient had a complication, serious intraoperative haemorrhage, which required a blood transfusion. The median follow-up time among 8 patients with recurrences was 43 months (ranging from 9 to 70 months). Relapse-free survival (RFS) was higher in the surgery group (p = 0.023, Fig. 3) in all patients, but there was no important difference in Overall survival (OS)(p = 0.772). Subsequently, we discovered that there was no statistical significance in overall survival (OS) between the partial gastrectomy group and the total gastrectomy group in the surgical cohort (p = 0.218, Fig. 4).

### Univariate and multivariate analysis

First, we performed a logistic regression to reveal risk factors for tumor recurrence between patients in surgery and ESD for curative resection groups. Univariate analysis identified the ESD group(p = 0.034), and the main lesion size > 2 cm(p = 0.019) with statistically significant for the risk of tumor recurrence. Then we included factors with P < 0.1 from univariate analysis into multivariate analysis, similarly, ESD group(p = 0.017), and the main lesion size > 2 cm(p = 0.011) were statistical significance (Table 2). Since only 1 patient in the surgery group had metastasis and died, we further explored the risk factors of tumor recurrence in the ESD for curative resection group by performing a logistic regression. In univariate analysis, main lesion size > 2 cm(p = 0.016), and minor undifferentiated lesion(p = 0.027) were statistically significant (Table 3). Multivariate analysis suggests that the important risk factor for tumor recurrence was the main undifferentiated lesion(p = 0.022).

## Discussion

In recent years, the diagnostic rate of early gastric cancer and gastric dysplasia has increased in China [1], due to the advances in endoscopic techniques and the popularity of gastric cancer screening. With these improvements in diagnosis, endoscopic treatment of early gastric neoplastic lesions without lymphovascular invasion has become more widespread. It is precisely because ESD preserves most of the stomach that the incidence of metachronous tumors after ESD is higher than that after gastrectomy [13], because of the propensity of the mucosa to develop multiple areas of potential dysplasia in selected patients. This is especially true for patients with multiple early gastric carcinomas in whom the appropriate treatment remains controversial. In our study, we compared the clinical prognostic outcomes between the surgery group and ESD for curative resection group. Our results revealed that the surgery group had a better RFS outcome than ESD for curative resection group. As expected, the complications in the surgery group were much higher than in the ESD for curative resection group(Table 4). We also found that the complications in partial gastrectomy group were decreased compared to those who underwent total gastrectomy. Notably, our univariate and multivariate analysis demonstrated that the main lesion size > 2 cm, and undifferentiated tumor were independent risk factors for tumor recurrence. Special attention should be given to this group of patients, such as regular endoscopy. After careful consideration, either radical distal gastrectomy or function-preserving gastrectomy may be considered as appropriate treatments.

According to previous studies, older age, male sex, well-differentiated tumors, a family history of gastric cancer, smoking, and drinking were important risk factors for multifocal gastric cancer [14, 15]. Nitta et al. reported that age ≥ 65 years and severe intestinal metaplasia of surrounding mucosa were important independent risk factors for multifocal gastric cancer by multivariate analysis [3]. Our findings are similar to prior research, most (79%,111/140) of patients in groups were male, and 62% (87/140) of the patients were with age ≥ 65 years. The main lesion in 81(58%) patients were diagnosed with well-differentiated gastric cancer, and 28(20%) patients were diagnosed with dysplasia. One of the possible reasons is that most of the patients were early gastric carcinoma in this study. Hereditary diffuse gastric cancer (HDGC) is a dominantly inherited cancer syndrome characterized by a high incidence of diffuse gastric cancer [16]. Several studies have shown that HDGC is closely related to the germline pathogenic variation of E-cadherin gene (CDH1) [17, 18]. At present, CDH1 variant carriers diagnosed with HDGC should be advised to undergo prophylactic total gastrectomy [19, 20]. However, data on whether our patients had a CDH1 mutation are not available because we were not able to evaluate them for this mutation; we understand that this is a limitation of our retrospective study lacking data in the CDH1 mutation. More recently we have become more aware of this possibility and are evaluating certain patients for this mutation, especially those with family members who have had gastric cancer.

Next, we compared the clinical outcomes and prognosis between ESD and gastrectomy in patients with multifocal gastric neoplasms and found that there existed no significant difference in OS. Additionally, the postoperative adverse events were significantly less in the ESD group than in the gastrectomy group. However, during the follow-up period, we found that the recurrence rates were higher in the ESD group, which is similar to the latest study by Xu et al. [9]. One probable explanation is that recurrence was detected during the patients’ postoperative gastroscopy, and timely surgical resection after diagnosis was performed [21].As shown in Fig. 1, additional radical gastrectomy and ESD was performed in 4 and 8 of the patients with curative resection, respectively. For patients with multifocal early gastric cancer, identifying important risk factors is urgently needed to reduce tumor recurrence rate.

Herein, our research investigated the risk factor for carcinoma recurrence between patients in gastrectomy and ESD for curative resection groups and we found that ESD and the size of main lesion > 2 cm are closely associated with higher risk of carcinoma recurrence. To further elucidate the causes of recurrence in patients undergoing ESD for curative resection, our study subsequently compared recurrent and non-recurrent patients in ESD for curative resection group. We found that the undifferentiated type of the main lesion will significantly increase the risk of carcinoma recurrence, which is consistent with our traditional views [22].

In regard to the location of main lesions, Chen et al. and Kim et al. reported that the location of the main lesion is mostly in the lower third [23–25]. Our data show that the location of the main lesion is mostly in the lower third, while the middle third has the least, which is consistent with previous study. In contrast, Nitta and colleagues reported that the middle third had 50 cases(54%), but their study still proved that more than half of the patients’ tumor location were located in the lower two-thirds of the stomach [3].

At present, the treatment of multifocal gastric cancer is similar to that of single gastric lesions, and still remains controversial. Kasuga et al. and Joh et al. reported that ESD is a feasible and effective option for the treatment of synchronous early gastric carcinoma [5, 26]. However, previous studies have shown that with ESD in the treatment of multifocal gastric cancer, there will be a high recurrence rate of tumors, as well as minor lesions missed with endoscopy [9, 27]. Therefore, Morgagni et al. found that subtotal gastrectomy could be considered a sufficient treatment when gastroscopy determines that the lesions of multifocal gastric cancer are limited to the lower third [25]. Within our study, there were 43 patients with total gastrectomy in the surgery group, and 10 patients had serious complications after surgery, while only 1 patient (1/30) had serious complications when underwent distal partial gastrectomy. In recent years, a number of studies have shown that distal gastrectomy has fewer complications compared to total gastrectomy [28, 29]. Notably, there was no significant difference in the survival rates of patients between the two groups, which is consistent with a previous study by Mocan et al. [29]. Therefore, we found that patients with distal partial gastrectomy have a better prognosis and no recurrence of the tumor. For multifocal gastric cancer patients with high risk factors for tumor recurrence, distal partial gastrectomy can be considered as adequate treatment.

We acknowledge that our study has certain limitations. First, this research was a retrospective study, which means that selection bias cannot be completely avoided. Second, we lack of genetic evaluation for e-cadherin positivity due to the limitation of the retrospective study. Thirdly, it is a single-center study with a limited sample size. Therefore, multicenter studies with larger sample sizes are required to confirm our findings.

## Conclusion

In conclusion, while the OS of ESD is comparable to surgery for curative treatment of SMEGC, ESD has a higher incidence of tumor recurrence compared to gastrectomy. Patients with SMEGC who have a main tumor size ≥ 2 cm and undifferentiated type are at higher risk of recurrence and require more attention. Regular endoscopic monitoring of patients with these characteristics is necessary. Additionally, we found no significant difference in OS between partial and total gastrectomy in the surgical cohort. Therefore, for SMEGC patients with high risk factors for tumor recurrence and limited lesions in the lower third, partial gastrectomy may be considered as adequate treatment.

## Data Availability

The raw data in this study can be obtained from the corresponding author on reasonable request by email(mastctl@163.com).

## Abbreviations

SMEGC: Synchronous multiple early gastric cancer
ESD: Endoscopic submucosal dissection
RFS: Relapse-free survival
OS: Overall survival
HDGC: Hereditary diffuse gastric cancer
